# Hydatid cyst of the interventricular septum

**DOI:** 10.21542/gcsp.2017.9

**Published:** 2017-03-31

**Authors:** Endale Tefera, Joseph Knapp, Michael Teodori

**Affiliations:** 1 Department of Pediatrics & Child Health, Cardiology Division, School of Medicine, Addis Ababa University, Addis Ababa, Ethiopia; 2 International Heart Institute of Montana, Missoula, Montana, USA; 3 Department of Surgery, Pediatric and Adult Congenital Heart Surgery division, University of Arizona Tucson, AZ, USA

## Abstract

While cardiac involvement is not a common presentation in human echinococcosis, it may lead to life-threatening complications including cyst rupture; anaphylactic shock; tamponade; pulmonary, cerebral or peripheral arterial embolism; acute coronary syndrome; dysrhythmias; infection; ventricular or valvular dysfunction, as well as sudden death. Here we report a 9-year old girl who was diagnosed to have hydatid cyst of the interventricular septum four years after diagnosis and medical treatment of pulmonary hydatidosis. Presentation, management and follow-up of the patient is discussed.

## Case presentation

A 9-year old girl was seen at a pediatric cardiac clinic of a university hospital for complaints of palpitation and decreased exercise tolerance of around two years duration. History of past illness revealed that the girl was admitted for chronic cough and pulmonary right lung mass at the age of 5 years. Her family report that a CT scan of her chest showed a right lung mass on that admission. They were told that she will undergo thoracotomy but later were told that medical treatment was sufficient (detailed record not available). On physical examination, She weighed 34 kg and her height was 149 cm. Her blood pressure was 121/53 mmHg and her pulse rate was 72 beat per minute. Her saturation was 93% at room air. She demonstrated no pallor or icterus. The chest was clear with good air entry. Peripheral pulses were palpable and there was no precordial bulge. No heave or thrill was noted. Point of Maximum Intensity (PMI) was in the 5th intercostal space along the left mid clavicular line. There was no murmur or gallop. There was no organomegaly or peripheral edema.

**Figure 1.**
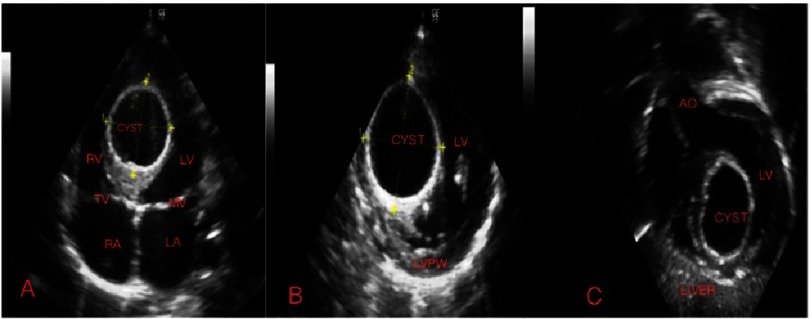
A. Echocardiographic frame in an apical 4-chamber view showing a large cystic mass splitting the interventricular septum. B. Parasternal short axis view, C. Subcostal coronal view.

Frontal chest X-ray performed at the time of current presentation showed no lung parenchymal abnormality, no pleural effusion and normal cardio-thoracic ratio and normal cardiac borders. Electrocardiography showed sinus rhythm with 80 beats per minute without any remarkable abnormality. A transthoracic echocardiogram demonstrated a large cystic mass measuring 3.8 cm × 3.4 cm within the interventricular septum, bulging in to the left ventricle (Figure 1A–C). No cysts were seen in the liver, kidney or spleen. LV systolic function was reduced (left ventricular ejection fraction of 40% and fiber shortening fraction of 20%). Minimal mitral regurgitation was noted. There was no pericardial effusion.

**Figure 2.**
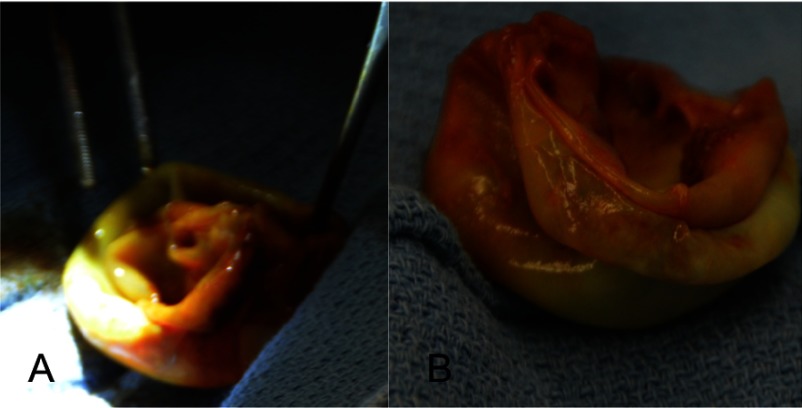
Hydatid cyst removed from the interventricular septum (A and B) cystic mass with double walls.

The patient was started on preoperative albendazole to be taken for 3-months. However, due to a misunderstanding during dispensing from the pharmacy, the patient took albendazole for only 3 days. As it was a mission-based surgery and she was symptomatic, we decided to proceed with surgical removal, in spite of suboptimal pre-surgical chemotherapy. Under cardiopulmonary bypass, the heart was arrested, and then opened via a vertical right ventriculotomy just right of the left anterior descending coronary artery. A large cyst of about 5 cm in size was identified in the interventricular septum. A betadine soaked sponge was placed in the right ventricle, and then the cyst was aspirated with a large bore needle. The cyst was then injected with concentrated iodine solution. The cyst was incised, suctioning away the contents while trying to avoid spillage into the heart and/or pulmonary arteries. The cyst was then excised completely (Figure 2A, B), opening the remaining cavity of the septum widely into the right ventricle. The right ventriculotomy was closed with a piece of extracellular matrix. Aortic cross-clamp time was 57 minutes and CBP was 82 minutes. She was extubated in the operating room but was in complete heart block and needed an external pacemaker. Hours after transfer to the ICU, the girl developed severe pulmonary edema with massive frothy secretions. Her blood gas parameters deteriorated within few hours. CXR suggested primarily a left lung injury. She was re-intubated and responded to 8 PEEP. She was started on inotropic agents and given bicarbonate. She showed progressive improvement and was successfully extubated on the third postoperative day. Her 3rd degree AV block resolved after several days. She then recovered nicely, and was started on albendazole to be taken for 18-months.

**Figure 3.**
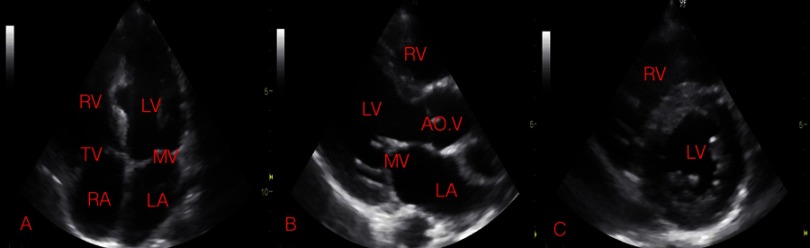
Echocardiographic frames taken 3-years after removal of hydatid cyst from the interventricular septum (A. 4-chamber view, B. Parasternal long axis view, C. Parasternal short axis view), with the only evident residual abnormality being a thinned area in the interventricular septum at the site of the prior cyst.

One month after the surgery, the girl was in a good clinical condition. She had good exercise capacity and the palpitations had resolved. Echocardiography showed no residual interventricular mass, no regional wall motion abnormality and no atrio-ventricular valve regurgitation. Her left ventricular systolic function normalized with an ejection fraction of 64% and fiber shortening fraction of 35%. Subsequent clinical condition and echocardiographic parameters were excellent. A two-dimensional echocardiogram taken three years after surgery, is shown in Figure 3A–C, with the only evident residual abnormality being a thinned area in the interventricular septum at the site of the prior cyst.

## Discussion

Cardiac involvement is not a common presentation of echinococcosis (0.5–2%).^1,2^ Clinical manifestations that result from cardiac hydatid cyst are dependent on the specific location of the cyst within the heart and the resulting interference with the function of the surrounding cardiac structures.^3^ However, majority of the patients with cardiac hydatidosis are asymptomatic.^4^ When symptomatic, the presentations include anaphylaxis; symptoms of low cardiac output (can act as a space-occupying lesion, as ventricular outflow obstruction, or as constrictive pericarditis); palpitation (due to irritation of conduction fibers in the ventricular septum); systemic or pulmonary emboli (rupture of the cyst in the cardiac chambers) or rapid and progressive pulmonary hypertension (due to embolization of high number of solices into the pulmonary circulation).^3,5,6^ Echocardiography is a preferred and efficient modality for the diagnosis of cardiac hydatidosis.^4,7^ Electrocardiographic abnormalities such as Q waves and inverted T waves in the inferior leads may also occur. When present, this signs should raise the suspicion of cardiac hydatidosis in patients living in endemic regions even when clinical symptoms are absent.^8^

Surgical treatment is the only option for cardiac hydatid disease since medical therapy does not offer assurance against rupture of the cyst and its potential complications.^9^ In the case of our patient, her pulmonary hydatidosis was not treated surgically. As a result, she presented with a more dramatic form (cardiac hydatidosis) after four years. We planned a 12-week preoperative albendazole therapy, but it was not practical because of a misunderstanding by the dispensing pharmacist. Some authors have suggested sterilization of the cyst content before manual handling using 2% formalin or hypertonic saline.^9^ In our case, we used direct instillation of concentrated iodine into the cyst.

Our patient developed pulmonary edema few hours after successful removal of the cyst from the interventricular septum. We tried to be meticulous about keeping cyst contents from spilling into the heart and/or pulmonary arteries, but it may be that some betadine solution drained dependently down the left pulmonary artery. She was successfully managed with modestly increased PEEP, and recovered from the pulmonary edema. We recommend placing a gauze sponge or Foley catheter with a 30 ml balloon inflated in the right ventricular outflow tract to prevent betadine or cyst content leakage into the distal pulmonary artery bed, to minimize the risk of lung injury. Though it has been reported in the literature that postoperative albendazole has been given for a duration as long as five years,^8^ our patient was treated with 18-months of albendazole after surgery.

In conclusion, hydatid cyst of the heart and specifically, the interventricular septum is rare. We are reporting this case to underline that cardiac hydatidosis should be considered as a differential diagnosis in patients who live in endemic regions presenting with an unexplained cardiac symptoms. The clinical history of our case also underlines that medical treatment alone may not be relied on to treat hydatidosis whether it is extracardiac or cardiac. Regarding surgical removal of the cyst, placing a gauze sponge or Foley catheter with a 30 ml balloon inflated in the right ventricular outflow tract may help to prevent betadine or cyst content leakage into the distal pulmonary artery bed, and minimize the risk of pulmonary injury.
